# Collectanea Medica: Consisting of Anecdotes, Facts, Extracts, Illustrations, &c.

**Published:** 1824-02

**Authors:** 


### COLLECTANEA MEDICA:

CONSISTING OF

### ANECDOTES, FACTS, EXTRACTS, ILLUSTRATIONS, &c.

***Relating to the History or the Art of Medicine, and the Collateral Sciences.***

---

Floriferis, ut apes, in saltibus omnia libant,
Omnia nos, itidem, depascimur aureâ dicta.

---

### ART. I.—*Extracts from "Medical Jurisprudence."* By J. A. PARIS, M.D. &c. and J. S. M. FONBLANQUE, Esq.

*Introduction.*—WE have at length arrived at the third, and most important, division of our work, comprehending the consideration of the principal pleas of the crown, three of which, Rape, Arson, and Murder, are pre-eminently the subjects of Medical Jurisprudence.

It is in the investigation of these crimes that the law derives its greatest support from the lights of science, and that the profession of physic demonstrates the value and extent of her judicial utility. Let the physician, then, who approaches the tribunal of justice, in order that he may promote by his science the due execution of the laws, fully appreciate the heavy responsibility of his situation;—let his evidence be so distinguished by its dispassionate and inflexible character, and his opinions be so matured by study and fortified by experiment, as not only to insure for himself the respectful attention of the court, but to afford a practical illustration of the just pretensions and importance of the liberal profession which he represents. The observations which we have already offered on the subject of medical evidence render it unnecessary for us to enlarge, on this occasion, upon the various duties it involves; and yet we cannot forbear from again pressing upon the attention of all those who are likely to be called upon to assist the ends of justice, the great importance of preparing their minds by preliminary studies: let it be remembered, that it is not during the hurry and anxiety of a coroner's inquest, nor amid the tumult of popular prejudice and execration, that a medical practitioner should, for the first time, adopt the physiological or chemical opinions by which he is ultimately to decide upon the life of a fellow creature: and yet it would be folly to conceal the unwelcome truth, that such a fact has not unfrequently occurred on several of the more interesting trials, upon which the medical witness has evinced any thing rather than a well-grounded acquaintance with the philosophical bearings of the question; and, while he has endeavoured to conceal his ignorance under the veil of technical phraseology, he has artfully sought to shun the embarrassments it might create by a display of bold and sweeping assertions, alike hostile to the discovery of truth and the administration of justice. There is yet another evil to which those who are but imperfectly informed on the question at issue are peculiarly exposed: their opinion is always liable to be warped by extraneous circumstances, and they are in consequence involuntarily apt to bend facts to their first view of the case under consideration, to seize on a few circumstances which suit their preconception, and to neglect or distort those which have a contrary tendency: while, on the other hand, the practitioner who has prepared his mind by study and experience, will, with equal diligence, seek every avenue to truth, and will suspend his conclusions until the result of each investigation be fairly before him. In delivering to the court the opinion to which his researches have led him, he will be ever careful to distinguish between the duties of an advocate, and those of an unbiassed witness; he will state whether the conclusion at which he has arrived amounts to certainty, or only to high probability, and will separate the doubts and difficulties with which the question has been encompassed by the sophistry of counsel, from those that belong intrinsically to the subject, and are inseparable from it. And it may be proper on this occasion to observe that the medical practitioner is not to withhold an opinion because it may be involved in doubt: he is to furnish the best evidence which the nature of the case will allow, and, when he duly performs this task, he may feel proud in the consciousness that he occupies an important station in the administration of justice; and that he conscientiously discharges a duty, without the due performance of which the laws of his country would be inoperative.

### *Arson.*

The charge of arson may occasionally become the subject of scientific research, and the accused individual receive an honourable acquittal at the hands of the chemical philosopher; by whose interposition, the conflagration, unjustly imputed to malice, may be proved to have originated from a spontaneous process of decomposition.

*Spontaneous combustion* may be defined, *an inflammation occasioned by the re-action of different bodies upon each other, at the ordinary heat of the atmosphere, without the contact or approach of any other body previously raised to a high temperature.* This definition necessarily excludes that class of substances which evolve gaseous matter of a highly inflammable nature, but which requires the appreach of an ignited body to kindle it.

The subject of spontaneous combustion has attracted the attention of many very eminent chemists, and an extensive series of experiments has been instituted in several different countries for its complete investigation, the results of which have thrown considerable light upon the causes which operate in the production of the phenomenon, as well as upon the nature of the substances most liable to such an accension, and the particular circumstances which are essential to its occurrence. The following may be considered as the principal sources from which it may originate,—viz.

I. *Friction.*

II. *Fermentation of Vegetable and Animal Substances,* as that of hay, oatmeal, roasted bran, coffee, &c. rags in paper-mills, &c.

III. *Chemical Action.* Accension of oils, by various animal, vegetable, and mineral substances; accension of vegetable matter by concentrated acids; ignition of lime by the affusion of water; ignition of pyrites.

We shall proceed to consider these subjects more in detail.

I. *Friction.*—The kindling of machinery, when not sufficiently greased, from the friction of its various parts, has occurred too frequently to require much illustration, although the immediate cause of the phenomenon involves in its consideration so many recondite points in the theory of caloric, as at present to elude our attempts at explanation; we must therefore rest upon it as an ultimate fact, and be satisfied with availing ourselves of the advantages to which a knowledge of it may conduce. The original inhabitants of the New World, throughout the whole extent from Patagonia to Greenland, procured fire by rubbing pieces of hard and dry wood against each other, until they emitted sparks, or kindled into flame; some of the people to the north of California produced the same effect by inserting a kind of pivot in the hole of a very thick plank, and causing it to revolve with extreme rapidity. This fact will explain how immense forests have been consumed, from the violent friction of the branches against each other by the wind.

II. *Fermentation of vegetable and animal substances.*—In order to establish the process of fermentation, the presence of water appears indispensable: we accordingly find that, in all the cases of spontaneous combustion which have originated from this source, the substances have either been in themselves imbued with moisture, or they have possessed the power of absorbing a considerable portion of water from the atmosphere. The firing of hay, when stacked in too moist a condition, is a striking exemplification of this fact. The same circumstance occurs from great accumulations of turf, flax, and hemp, heaps of linen rags in paper-mills, &c. provided a sufficient portion of moisture be present to excite the process of fermentation, and the consequent evolution of heat. Oatmeal, from the extreme avidity with which it imbibes water,* and the heat which is generated by the absorption of it, is necessarily liable to spontaneous combustion; the following well-authenticated case may serve as an illustration of this fact:

"A gentleman removed with his family from Glasgow to Largs, in May last, and shut up his house, which was not re-opened until the end of August. The house stands on the side of a steep declivity, so that the kitchen, which is in the back part, though sunk considerably below the level of the street, is entirely above ground, and is well lighted and ventilated. In an opening of the wall near the kitchen fire-place, originally intended, it is supposed, for an oven, there was placed a wooden barrel, bound with iron hoops, and filled with oatmeal. This meal, which had heated during the absence of the family, at last caught fire, and was totally consumed, together with the barrel which contained it; nothing remaining but the iron hoops and a few pieces of charcoal."†

In some cases torrefaction increases the propensity of vegetable substances to spontaneous combustion: coffee, roasted French beans, lentils, &c. are of this description. Some years ago a great fire broke out

---

* "Mr. Leslie has availed himself of this property in oatmeal, and has applied the substance in place of sulphuric acid, in his ingenious and beautiful experiment of freezing in the exhausted receiver of the air-pump.

† *Annals of Philosophy*, vol. xvi. p. 390.

in the village of Nauslitz, which is said to have been occasioned by the application of roasted bran to the necks of some cattle in a wooden cow-house; in consequence of which, M. Rude, an apothecary at Bautzen, instituted some experiments, by which he found that, if rye-bran, roasted until it acquires the colour of coffee, be wrapped up in a linen cloth, it will in a short time take fire. Montet relates* that animal substances may also, under certain circumstances of decomposition, kindle into flame; and he tells us that he had himself witnessed the spontaneous accension of a dunghill. We do not believe that the phosphoric appearances that so frequently accompany the process of putrefaction, especially that of fish, are ever connected with actual combustion. Woollen stuffs are said to have taken fire spontaneously: it is related, for instance, that the article manufactured at Cevennes, and which bears the name of *Emperor's stuff*, has thus kindled of itself, and burnt to coal. We are, however, very doubtful whether such a material is liable to this process, unless it be impregnated with oily matter; and this doubt will receive considerable strength from the facts which we shall hereafter enumerate.

III. *Chemical action.*—This proves a very frequent cause of spontaneous combustion; and there is perhaps no substance that has so frequently performed the part of an incendiary as fixed oil, especially when of a drying nature, which, with its various accomplices from the animal, vegetable, and mineral kingdoms, has, in darkness and secresy, consigned ships, houses, and manufactories to the flames. The following occurrence is related in the Edinburgh Philosophical Journal:—"About twenty-five pieces of cloth, each of which contained nearly thirty ells, were deposited upon wooden planks in a cellar at Lyons, on the 8th of July, 1815, in order to conceal them from the armies which then over-ran France: in the manufacture of the cloth, 25lbs. of oil were used for a quintal of wool, and the cloth was quite greasy, each piece weighing from 80lbs. to 90lbs.; the cellar had an opening to the north, which was carefully shut up with dung, and the door was concealed by bundles of vine-props, which freely admitted the air. On the morning of the 4th of August an intolerable stench was perceived, and the person who entered the cellar was surrounded by a thick smoke, which he could not support. A short time afterwards he re-entered with precaution, holding a stable lanthorn in his hand, and he was astonished to perceive a shapeless glutinous mass, apparently in a state of putrefaction: he then removed the dung from the openings, and, as soon as a circulation of air was established, the cloth instantly took fire. In another corner of the cellar lay a heap of stuffs, which had been ungreased and prepared for the fuller, but *they had suffered no change.*" In this case the agency of the oil was sufficiently evident.

In June, 1781, a similar occurrence happened at a woolcomber's, in a manufacturing town in Germany, where a heap of wool-combings, piled up in a close warehouse seldom aired, took fire spontaneously. This wool had been by little and little brought into the warehouse, and, from want of room, been piled up very high and trodden down. That

---

* *Memoires de l'Academie de Paris*, 1743.

this combed wool, to which rape-oil mixed with butter had been added in the combing, burnt of itself, was sworn to by many witnesses; one of whom affirmed that, ten years preceding, a similar fire had happened among the flocks of wool at a clothier's, who had put them into a cask, where they were rammed down hard for facility of carriage, and that this wool burnt from within outwards, and became quite a cinder.

Cotton goods, in which linseed-oil had been spilt, have burnt in a similar manner, and there is reason to attribute to an accident of this kind the recent loss of a merchant-vessel, homeward bound from the East Indies.

Many years since, several fires broke out, at very short intervals, in a rope-walk, and in some wooden houses in St. Petersburgh; in none of which instances could the slightest suspicion of wilful firing be entertained. There was lying in the rope-walk, where the cables for the navy are made, a great quantity of hemp, amongst which a considerable portion of oil had been carelessly spilt, and the article was accordingly declared to have been spoilt; in consequence of which it was purchased at a low price, and, being heaped up together, it had given rise to the conflagration. The inferior inhabitants had also purchased parcels of this spoilt hemp, for closing the chinks and caulking the windows of their houses; a fact which offered an easy explanation of the origin of the fires that occurred amongst the houses. It was moreover reported that, at the above-mentioned rope-walk, coils of cable had been frequently discovered so hot, that the people were obliged to separate them to prevent further danger. In the year 1757, as Montet reports, sail-cloth, smeared with oil and ochre, took fire in a magazine at Brest. In the spring of 1780, a fire was discovered on-board a frigate lying in the road off Cronstadt, which, had it not been timely extinguished, would have endangered the whole fleet. After the most severe scrutiny, no cause of the fire was to be found, and strong surmises existed that some wicked incendiary had occasioned it.

It sometimes happens that, in boiling flowers and herbs in oil, which occurs in several pharmaceutic operations, these herbs, after being taken out, dried, and pressed, inflame spontaneously: care, therefore, should be taken, when such substances are thrown aside, that they are not heaped up near other combustible bodies.

Amongst the mineral substances capable of exciting the inflammation of oils, an ore of manganese, known by the name of the *black wad of Derbyshire*, holds a distinguished place: when this substance is pulverised, and moistened with a little linseed-oil, it will, in the space of an hour, take fire, and become red hot, like burning small-coal. It is supposed that the Pantheon, in Oxford-street, was destroyed by the inflammation of a compound of Derbyshire wad and oil, used in painting the scenery.

In these cases of combustion, oxygen seems to act an important part, and, by combining with the hydrogen of oil, to excite a chemical action, which may be considered the immediate cause of the phenomenon. Saw-dust, and other vegetable matter, has been occasionally excited into flame by the action of the concentrated mineral acids. We have been lately informed by Mr. Parkes, that a fire took place, some years since, in his chemical manufactory, in consequence of the leakage from a carboy of nitric acid. Several instances are also on record of fires having been occasioned by the sudden slaking of quicklime. Theophrastus relates an instance of a ship which was loaded in part with linen, and in part with quicklime, having been set on fire by water that was accidentally thrown over the latter, and that the vessel was in consequence entirely consumed. In the Journal de la Haute Saone, there is an account of the burning of a barn, one of the partitions of which, being wood, had caught fire from a quantity of quicklime, intended for the repair of the premises, having been carelessly thrown against it. In this country, a similar accident happened in the last winter at Edmonton, near London: the flood, consequent upon a heavy fall of rain, made its way among the quicklime in a bricklayer's premises, which took fire, and were burnt.

There still remains for notice another source of spontaneous burning,—the ignition of *pyrites*, and that of cinders from the furnaces of glass works, from exposure to air and moisture. It was in this manner that the ship Ajax was supposed to have been consumed, from the spontaneous combustion of coal abounding in pyrites.

### *Human Combustion.*

Before we quit the consideration of spontaneous combustion, it becomes our duty to offer a few observations upon a subject which appears to be nearly allied to it, and which certainly belongs to medico-judicial inquiry,—*the combustion of human beings.* The phenomenon, however, has been erroneously designated as *spontaneous;* for, in every recorded instance, the approach of some burning body, as that of the flame of a candle or an ignited pipe, appears to have been necessary for its occurrence. "It can no longer be doubted," says Dr. Gordon Smith, "that persons have retired to their chambers in the usual manner, and in place of the individual a few cinders, and perhaps part of his bones, were found." Upon this occasion we confess ourselves more sceptical: the phenomenon is contrary to all our preconceived views, and must therefore require more than ordinary testimony for its support; although we are ready to admit that, upon any other less miraculous subject, evidence even less powerful than that produced on the present occasion, would be deemed amply sufficient. Plouquet, in his Literatura Medica, enumerates twenty-eight cases. Dr. Trotter, in his Essay on Drunkenness, adduces a considerable number of instances of persons, addicted to the immoderate use of spirits, having undergone such combustion. In Paris, an essay written exclusively on this subject was published by Pierre Aimée Lair, entitled "Essai sur les Combustions Humaines, produites par l'abus des liq. spirit: Paris, 1808;" and the Journals of various nations present us with a great variety of examples, all of which, with some slight shades of difference, appear to have been attended with the same phenomena; a fact which, we freely admit, affords internal evidence of their authenticity. On the other hand, it deserves notice that, amidst all these cases, *only one*

is related where the person survived for a short time, and gave an account of the manner in which he was *struck* with the fire: in none of the others has it ever been known in what way the fire commenced, or proceeded.

The following are the circumstances in which all the recorded cases so singularly concur:

1. The persons who have suffered this species of combustion have been long accustomed to drink spirituous liquors.

2. These persons have been generally females, and advanced in years.

3. The body has not burned spontaneously, but accidentally, in as much as it required for its inflammation the contact or approach of some burning body, or that of electric matter.

4. The extremities of the body, such as the feet and hands, have in general escaped.

5. The fire has little injured, and sometimes not at all, those combustible things that were in contact with the body when it was burning.

6. The combustion of these bodies has left a residue of greasy and fetid ashes and fat, that were unctuous, and extremely offensive and penetrating.

Various theories have been proposed for the explanation of this singular phenomenon; and we may here observe, that, if the bodies in question were actually found consumed in the manner described, it is quite impossible to suppose that they were burnt by ordinary means; nor, even admitting that they had been rubbed over with a highly combustible substance, is the explanation less difficult. At a period when criminals were condemned to expiate their crimes in the flames, it is well known what a large quantity of combustible materials was required for burning their bodies. A baker's boy, named Renaud, being several years ago condemned to be burnt at Caen, two large cart-loads of faggots were required to consume the body; and at the end of more than ten hours some remains were still visible. In this country, the extreme incombustibility of the human body was exemplified in the case of Mrs. King, who, having been murdered by a foreigner, was afterwards burnt by him; but in the execution of this plan he was engaged for several weeks, and after all did not succeed in its completion.

